# Mental health outcomes and use of online mental health tools following a public health deployment of the mindfulness meditation app headspace

**DOI:** 10.3389/fdgth.2026.1793455

**Published:** 2026-05-07

**Authors:** Judith Borghouts, Elizabeth V. Eikey, Cinthia De Leon, Jeongmi Kim, Stephen M. Schueller, Margaret Schneider, Nicole A. Stadnick, Kai Zheng, Dara H. Sorkin

**Affiliations:** 1 Department of Medicine, University of California, Irvine, CA, United States; 2 Herbert Wertheim School of Public Health and Human Longevity Science, University of California, San Diego, CA, United States; 3 The Design Lab, University of California, San Diego, CA, United States; 4 Department of Informatics, University of California, Irvine, CA, United States; 5 Department of Psychology, University of California, Irvine, CA, United States; 6 Department of Public Health, University of California, Irvine, CA, United States; 7 Department of Psychiatry, University of California, San Diego, CA, United States; 8 Dissemination and Implementation Science Center, UC San Diego Altman Clinical and Translational Research Institute, La Jolla, CA, United States; 9 Child and Adolescent Services Research Center, San Diego, CA, United States

**Keywords:** digital mental health interventions, headspace, mental health, mental health resources, mHealth, mindfulness

## Abstract

**Background:**

Digital mental health interventions (DMHIs), such as mindfulness-based apps, have become popular mental health resources and can be associated with benefits such as reducing stress, anxiety, and depression. However, there are limited data on the impact of DMHIs on improving mental health outside of controlled research settings, and what factors may facilitate or inhibit such potential impact. This study aimed to understand whether use of Headspace, a mindfulness meditation app, within the context of a large-scale public deployment affected mental health outcomes, mental health stigma, and utilization of other mental health resources.

**Methods:**

All community members who received Headspace as part of the deployment (*N* = 97,709) were invited to complete an initial survey (time point 1). A second survey (time point 2) was administrated 4 weeks after. Both surveys were voluntary and uncompensated. Participants (*N* = 1,229) who had used Headspace and who completed both surveys were included in the analysis. Regression models were used to examine relationships between frequency and continued use of Headspace as well as experiences with Headspace, and changes in mental health problems, mental health stigma, and mental healthcare utilization between time points 1 and 2.

**Results:**

There was a significant decrease in distress and loneliness over time, regardless of how long or how frequently the participants used Headspace. Those who continued to use Headspace had a larger decrease in mental health stigma resistance compared to those who stopped using Headspace after time point 1. Additionally, those who stopped using Headspace were more likely to use other online mental health tools at time point 2 compared to those who continued to use Headspace.

**Conclusions:**

Taking part in a program that provides access to a mindfulness app like Headspace may be beneficial for those in need of mental health support. Additionally, people who moved on from Headspace may have already had or found other digital mental health resources that worked well or better for them.

## 1 Introduction

 Mental health remains a critical public health issue. Various barriers can keep individuals from accessing the right resources, such as cost, stigma, and accessibility (1). In response, digital mental health interventions (DMHIs) have been proposed as a cost-effective and accessible way to offer mental health services (2). The development of DMHIs has vastly accelerated in recent years: as of 2023, over 10,000 mental health apps were available online (3). A popular feature supported among DMHIs is mindfulness meditation (4): an analysis of DMHIs found that mindfulness was the third most common feature, with 38% of analyzed products containing mindfulness features (5). Mindfulness refers to a state of nonjudgmental awareness of one's present experience. Mindfulness meditation is a practice dedicated to cultivating this state of awareness, and this practice has been shown to have positive effects on mental health and well-being (6). However, while the effect of DMHIs has been well-researched in controlled research settings with short timespans and small samples, there is limited data on how they are used in real-world settings. Specifically, there is a knowledge gap in whether DMHIs introduced through broad public deployments can achieve anticipated benefits in improving mental health over time, and what factors may contribute to their real-world impact. Further, it is important to identify what individual-level factors impact outcomes of such DMHI interventions in order to inform the design and implementation of future programs.

The current study evaluates a California-based innovation program, which offered Headspace, a DMHI for mindfulness meditation, as a free mental health resource to the communities. The study focuses on Headspace because it best met the needs and requirements of the innovation program in which it was implemented, such as privacy and security compliance, and availability of content in multiple languages. Additionally, Headspace is one of the most widely used DMHIs in the world (Headspace and Calm represent approximately 90% of DMHI users) (7), making it a valuable app through which to better understand DMHI use overall.

The aim of the study was to understand whether the experience with and use of Headspace affected mental health outcomes, mental health stigma, and utilization of other mental health resources besides Headspace, within the context of the program. Using findings from two surveys conducted four weeks apart, we were also able to examine changes in mental health and use of Headspace and other mental health resources over time.

### 1.1 Selection of study measures

The study focused on the outcomes distress, loneliness, mental health stigma, mental healthcare utilization and user experience for a number of reasons.

First, distress, loneliness, and mental health stigma are key dimensions of mental health that may be improved through DMHIs (8–10). Second, loneliness, stigma, and mental healthcare utilization aligned with the innovation program's objectives to improve social connectedness, reduce mental health stigma, and increase access to support and care. Third, distress, stigma, and mental healthcare utilization were also chosen to enable comparison with the California Health Interview Survey (CHIS) (11), the largest statewide health survey in the United States. This allowed the program to compare participant outcomes against broader state-level trends over time, though comparison findings are outside of the scope of the current paper. Fourth, collecting data from a large-scale deployment required balancing the views and priorities of multiple stakeholders, and we worked closely with participating counties and cities to develop the survey and select appropriate measures informed by both literature and the lived experiences of community members. This community-based approach helped ensure the measures were relevant to the community, fostered buy-in and supported survey participation and meaningful data. Lastly, user experience was included because the effectiveness of DMHIs in improving mental health outcomes is contingent on their use (12). Measuring user experience provided insight into whether lack of improvement or discontinued use was associated with a negative user experience.

Where possible, we used validated scales and survey questions. In response to stakeholder feedback, two validated scales were supplemented with positively worded items: one item was added to the loneliness scale (“How often do you feel connected with others?”) and one item was added to the distress scale (“During the last 30 days, about how often did you feel hopeful?”). These added items were not used in the calculation of the distress and loneliness scores and were excluded from the analysis. No original survey items were adapted. Further details on survey development, as well as internal consistency estimates for each measure are reported in Borghouts et al. (13).

## 2 Materials and methods

### 2.1 Program description

This study was part of Help@Hand, a state-wide innovation project in California that aimed to understand how DMHIs fit within the public mental health system of care. The project was funded and directed by several participating counties and cities (referred to as sites throughout the rest of the paper) in California and overseen by the California Mental Health Service Authority (CalMHSA).

### 2.2 Application background

Headspace is a mindfulness meditation app which offers content such as guided meditations, physical exercise videos, sessions to help with sleep, and music to help focus with work. The content is available in English, Spanish, French, German and Portuguese, and can be accessed through a mobile application, available on Android and Apple iOS, or the Headspace website.

Headspace was selected through a vetting process to ensure it would meet the needs of the Help@Hand project (e.g., to ensure that it had a privacy protocol in place to safely store and protect user data and that content was available in additional languages beyond English).

### 2.3 Study design and procedure

This paper reports on survey data collected between July 1, 2021 and November 28, 2023. Two surveys were administrated at two time points: 1) the initial survey (timepoint 1) was first sent on July 1, 2021 to all participants who had signed up for Headspace up until that point, and was sent once a week after that to all new participants who signed up that week, and 2) the follow-up survey (timepoint 2) was sent to participants four weeks after they completed the first survey. The four-week interval between the two surveys was designed to give participants sufficient time to use Headspace—consistent with prior work showing symptom improvements within 4 weeks (14, 15)—while aiming to still timely capture those who may have stopped using Headspace after timepoint 1.

Participants were sent an email with a unique link to complete a survey; the survey responses were anonymous and not linked to the participant's email address. The surveys were hosted on REDCap (16, 17), a secure, web-based software platform designed to support data capture for research studies. The surveys were available in English and Spanish, and survey completion was voluntary and unpaid.

97,709 participants received the first survey. Of them, 3,724 started the survey (response rate 3.8%, 3,724/97,709) and 2,995 completed it (completion rate 3.1%, 2,995/97,709). 1,455 participants started the second survey (response rate 48.6%, 1,455/2,995) and 1,332 completed it (completion rate 44.5%, 1,332/2,995).

The majority of people who completed the first but not second survey were between 26 and 59 years old (81.1%), identified as woman/female (70.4%), self-reported to be a current user at the time of the first survey (74.6%), and 73.6% identified they were experiencing mental health challenges. These characteristics are broadly similar to the larger sample, and there are no distinct trends distinguishing this group from those who completed both surveys.

**Figure 1:**
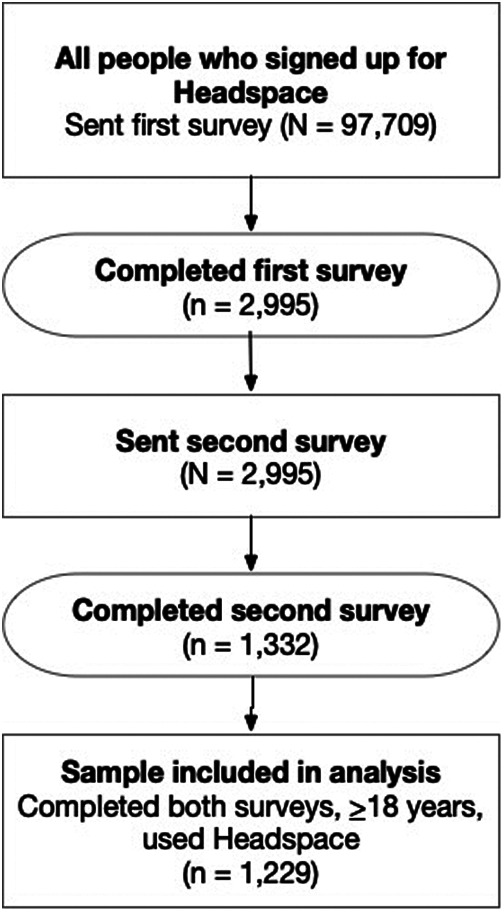
Flow diagram of included participants.

An analysis of participants who completed the first survey is reported in Borghouts et al. (13). The current paper focuses on a dataset of 1,229 participants who completed both surveys, were 18 years or older, and indicated they had used Headspace. Figure 1 shows a flow diagram of the included participants.

### 2.4 Ethical considerations

The study was approved by the University of California, Irvine, Institutional Review Board (20195406). Respondents were provided with a study information sheet approved by the institutional review board at the start of the survey, and had the option to download a copy. All research data were stored securely and confidentially on a password-protected secure server. Program participants were informed at the time of program enrollment that their email address would be shared with the research team and that they would be contacted to participate in a survey. Completing or not completing the survey had no impact on people's access to Headspace.

### 2.5 Measures

A community-based participatory approach was used to include community members in development of the survey instrument. This process is described in more detail in Borghouts et al. (13). The survey instrument is available as Supplementary Material online. A brief description of each measure is given below.

#### 2.5.1 Adoption of headspace

Respondents were asked one multiple-choice question on whether they were using Headspace at the time of completing the survey. Respondents could choose from the following answers: “Yes', “No, but I did use it in the past”, or “No, and I never used it”. In this paper, we only look at participants who said they were currently using it and/or had used it in the past. We will refer to those who indicated they were using Headspace as Current Users, and people who were no longer using Headspace as Past Users. Based on respondents' answers on the two surveys, we grouped respondents into four user types (see Table 1), which we labelled Continued Users (Current Users at both timepoints), Former Users (Past Users at both timepoints), Discontinued Users (Current User at timepoint 1, Past User at timepoint 2), and Resumed Users (Past User at timepoint 1, Current User at timepoint 2).

**Table 1:** An overview of individuals who were using Headspace (current users) and were no longer using Headspace (past users) at the time of survey 1 and 2 (*N* = 1,229).

Survey 1	Survey 2	Label	Sample, *n* (%)
Current User	Current User	Continued users	950 (77.30)
Past User	Past User	Former users	143 (11.64)
Current User	Past User	Discontinued users	64 (5.21)
Past User	Current User	Resumed users	72 (5.86)

#### 2.5.2 Frequency of Headspace use

Respondents were asked one question on how frequently they used Headspace. They could choose from the following answers: “Daily”, “Several times a week”, “Several times a month”, “About once a month”, “I only used it once”, or they could write in an answer.

#### 2.5.3 Distress

The Kessler Psychological Distress (K10) scale (18) was used to measure psychological distress. Respondents were asked to rate ten statements related to how they had been feeling during the past 30 days (e.g., “During the past 30 days, how often did you feel tired out for no good reason?”). The statements were rated on a 5-point Likert scale ranging from None of the time (1) to All of the time (5), with a total sum score in the range of 10–50. A higher score indicates a higher level of psychological distress.

#### 2.5.4 Loneliness

Loneliness was measured using the Three-Item Loneliness Scale (19), a shortened version of the University of California, Los Angeles Loneliness Scale (20). Respondents were asked to rate three statements related to their perceived loneliness (e.g., “How often do you feel left out?”) on a 3-point Likert scale ranging from Hardly ever (1) to Often (3) with a total sum score ranging from 3 to 9. A higher score indicates a higher degree of perceived loneliness.

#### 2.5.5 Mental health stigma

We measured four types of mental health stigma: internalized stigma, perceived stigma, help-seeking stigma, and stigma resistance. Items were taken from Internalized Stigma of Mental Illness Inventory (ISMI) (21) for internalized stigma and stigma resistance, the Perceived Stigma subscale of the Depression Stigma Scale (22) for perceived stigma, and the RAS-R (23) for resilience/help-seeking stigma. One item was chosen from each of these four subscales instead of all subscale items, based on feedback from community stakeholders to keep the survey brief and balance positively and negatively worded items (13), as well as key individual feedback from a 2019 in-person workgroup focused on stigma (24) and a subsequent factor analysis of responses from 6,304 participants.

These items asked respondents to rate four statements on a 5-point Likert scale from Strongly disagree (1) to Strongly agree (5): “Being around people who don’t have mental health challenges makes me feel out of place or inadequate” (internalized stigma), “Most people believe that having mental health challenges is a sign of personal weakness” (perceived stigma), “I know when to ask for help” (help-seeking stigma), and “In general, I am able to live life the way I want to” (stigma resistance).

#### 2.5.6 Mental healthcare utilization

Respondents were presented with four dichotomous (yes or no) response items adapted from the California Health Interview Survey (11) on whether they had used online tools other than Headspace for problems related to mental health, whether they had connected online with others with similar mental health problems, whether they had used online tools to connect to a mental health professional, and whether they had seen a mental health professional in the past 12 months (timepoint 1) or the past 30 days (timepoint 2).

#### 2.5.7 User experience

We measured respondents' user experience with Headspace using 12 survey items based on the Unified Theory of Acceptance and Use of Technology (UTAUT) questionnaire (25) designed to evaluate technology acceptance and adoption. Respondents were asked to rate 12 statements (e.g., “I find Headspace useful in my daily life”) on a 5-point Likert scale ranging from Strongly disagree (1) to Strongly agree (5). The items were combined into a single mean score that ranged from 1 to 5; a higher score indicates a more positive experience with Headspace.

### 2.6 Statistical analyses

Data were analyzed using statistical software R (version 4.2.2) (26). Paired t-tests were used to test for significant differences in distress, loneliness, mental health stigma, user experience, and frequency of use between timepoint 1 and timepoint 2. Chi-squared tests were used to test for significant differences in mental healthcare utilization between timepoint 1 and timepoint 2. A linear regression model was used to study the effect of Headspace use (timepoint 1 and 2), user experience (timepoint 1 and 2), and distress (timepoint 1) on distress (timepoint 2). A separate linear regression model was used to study the effect of Headspace use (timepoint 1 and 2), user experience (timepoint 1 and 2), and mental health stigma (timepoint 1) on mental health stigma (timepoint 2). A logistic regression model was used to study the effect of Headspace use (timepoint 1 and 2), user experience (timepoint 1 and 2), distress (timepoint 1), mental health stigma (timepoint 1), and mental healthcare utilization (timepoint 1) on mental healthcare utilization (timepoint 2). To be aligned with the other stigma items, help-seeking stigma and stigma resistance were reverse coded, such that higher scores equal more stigma. Therefore, we will refer to these constructs as “stigma towards help-seeking” (the negatively-worded version of help-seeking stigma) and “impact of stigma on life” (the negatively-worded version of stigma resistance). Age, gender, race and education level were included as covariates. Site (that is the participating county or city in which Headspace was offered) and length of Headspace use were added as control variables to make sure that length of use and any variations between sites did not affect the results.

## 3 Results

### 3.1 Participants

Table 2 shows respondents' demographic characteristics (*N* = 1,229). The majority of them were between 26 and 59 years old (997/1,229, 81.1%), identified as woman/female (912/1,229, 74.2%), and were highly educated with a bachelor's or master's degree (887/1,079, 82.2%). 608 (49.5%) identified as Non-Hispanic White, 208 (16.9%) identified as Hispanic/Latino/a/x, and 157 (12.8%) identified as Asian. At timepoint 1, 901 (73.3%) respondents indicated they experienced mental health challenges. There were no significant differences between user groups in demographics shown in Table 2, which were measured at timepoint 1.

**Table 2:** Demographics of participants (*N* = 1,229).

Demographics	*N*	%^a^
Age		
18–25	114	9.3%
26–59	997	81.1%
60+	118	9.6%
Gender		
Woman/Female	912	74.2%
Man/Male	265	21.6%
Other Gender Identity	52	4.2%
Race/Ethnicity		
Non-Hispanic White	608	49.5%
Hispanic/Latino/a/x	208	16.9%
Asian	157	12.8%
Black or African American	53	4.3%
Other Race/Ethnic Identity	203	16.5%
Highest Education Level		
Less than a Bachelor's Degree	209	17.0%
Bachelor's Degree	516	42.0%
Graduate or Professional Degree	483	39.3%
Language they used Headspace in		
English	1,024	94.1%
Spanish, Portuguese, German or French	22	1.8%

a Not all respondents answered each question; hence why some percentages do not sum up to 100%.

### 3.2 Changes in distress, loneliness, stigma and mental healthcare utilization over time

Table 3 shows a high-level summary of results by user type. Paired t-tests (Table 4) showed that distress scores decreased significantly from timepoint 1 to timepoint 2, t(1,219) = 2.63, *P* = 0.01. Loneliness also decreased over time, *t*(1,223) = 2.13, *P* = 0.03. Internalized stigma increased from timepoint 1 to timepoint 2, *t*(1,133) = −2.17, *P* = 0.03. It is important to note that effect sizes of these changes were small (Cohen's *d*s < 0.06), which is similar to prior large deployments of mindfulness-based mobile apps (27). Given the large sample size (*N* = 1,229), the study was sufficiently powered to detect effects far smaller than those typically considered meaningful, highlighting the importance of interpreting the magnitude rather than the statistical significance of these changes.

**Table 3:** A summary of results divided by user type (*N* = 1,229).

Survey 1	Survey 2	Label	Summary of results
Current user	Current user	Continued users	—
Past user	Past user	Former users	Lower help-seeking stigma scores at timepoint 2 compared to Continued Users
Current user	Past user	Discontinued users	Greater increase in impact of stigma on life at timepoint 2 compared to Continued Users
			More likely to engage with other online mental health tools at timepoint 2 compared to Continued Users
Past user	Current user	Resumed users	—
—	—	Full sample	Significant decreases in distress and loneliness, and a significant increase in internalized stigma, regardless of user type

**Table 4:** Paired t-tests comparing outcomes at timepoint 1 and 2.

Outcome	Timepoint 1 M (SD)	Timepoint 2 M (SD)	t	df
Psychological distress	21.5 (7.6)	21.2 (7.3)	2.63**	1,219
Loneliness	5.5 (1.9)	5.47 (1.8)	2.13*	1,223
Mental health stigma				
Internalized stigma	2.46 (1.4)	2.55 (1.3)	−2.17*	1,133
Perceived stigma	2.98 (1.3)	3.04 (1.2)	−1.63	1,210
Impact of stigma on life (reverse coded stigma resistance)	2.11 (1.1)	2.10 (1.1)	−0.48	1,206
Stigma towards help-seeking (reverse coded help-seeking stigma)	2.15 (1.2)	2.15 (1.1)	−0.12	1,207
User experience	3.63 (1.2)	3.63 (1.2)	0.35	1,209
Frequency of use	4.14 (0.7)	4.13 (0.7)	−0.12	1,192

* *P* < .05.

** *P* < .01.

Perceived stigma, stigma resistance (impact of stigma on life), and stigma towards help-seeking (help-seeking stigma) did not significantly change from timepoint 1–2, *P*s > .05. There were no significant changes in user experience or frequency of Headspace use, *P*s > .05.

Chi-squared tests (Table 5) showed that use of mental health resources also declined between timepoint 1 and 2: participants were less likely to report using online tools, *χ*^2^(1) = 163.45, *P* < .001; connecting with others online, *χ*^2^(1) = 240.95, *P* < .001; using online tools to connect to a professional, *χ*^2^(1) = 159.25, *P* < .001; and seeing a mental health professional, *χ*^2^(1) = 8.01, *P* = .005.

**Table 5:** Chi-squared tests comparing mental healthcare utilization outcomes at timepoint 1 and 2.

Outcome	Timepoint 1 *N* (%)	Timepoint 2 *N* (%)	*χ* ^2^	df
Mental healthcare utilization				
Online tools	527 (42.9%)	287 (23.4%)	163.45***	1
Connecting with people online	369 (30%)	314 (25.5%)	240.95***	1
Connecting with a mental health professional online	492 (40%)	312 (25.4%)	159.25***	1
Use of professional services	648 (52.7%)	471 (38.3%)	8.01**	1

** *P* < .01.

*** *P* < .001.

### 3.3 Predictors of distress, loneliness, stigma and mental healthcare utilization at timepoint 2

Regressions (Tables 6, 7) examined whether user type or frequency of use predicted outcomes at timepoint 2, controlling for baseline scores, demographics, and sites. Distress, loneliness, internalized stigma, and perceived stigma at timepoint 2 were not significantly predicted by user type or self-reported frequency of use, *P*s > .05.

**Table 6:** Linear regression results for the effect of user type on mental health and stigma outcomes. Reference group for user type is continued users (Current-Current users).

Outcome	Predictors	Estimate	SE	*t*-value	*R* ^2^	*N*
Psychological distress	Former users	−0.065	0.652	−0.099	0.60	1,151
	Resumed users	1.102	0.922	1.195		
	Discontinued users	0.630	0.931	0.676		
Loneliness	Former users	−0.172	0.165	−1.039	0.60	1,152
	Resumed users	0.209	0.234	0.896		
	Discontinued users	0.057	0.236	0.243		
Mental health stigma						
Internalized stigma	Former users	0.004	0.152	0.025	0.42	1,068
	Resumed users	0.210	0.212	0.991		
	Discontinued users	0.288	0.214	1.349		
Perceived stigma	Former users	−0.069	0.149	−0.463	0.28	1,142
	Resumed users	0.172	0.213	0.806		
	Discontinued users	0.000	0.211	0.001		
Impact of stigma on life (reverse coded stigma resistance)	Former users	−0.050	0.118	−0.420	0.39	1,141
	Resumed users	0.175	0.168	1.041		
	Discontinued users	0.375	0.167	2.251*		
Stigma towards help-seeking (reverse coded help-seeking stigma)	Former users	−0.273	0.117	−2.331*	0.47	1,142
	Resumed users	−0.185	0.167	−1.108		
	Discontinued users	−0.076	0.165	−0.461		

* *P* < .05.

**Table 7:** Logistic regression results for the effect of user type on mental healthcare utilization. Reference group for user type is continued users (Current-Current users).

Outcome	Predictors	Estimate	SE	*z*-value	OR	95% CI	*R* ^2^	*N*
Mental healthcare utilization								
Online tools	Former users	0.405	0.370	1.096	1.50	−0.335, 1.119	0.20	1,129
	Resumed users	−0.380	0.672	−0.565	0.68	−1.901, 0.814		
	Discontinued users	1.461	0.475	3.075*	4.31	0.508, 2.382		
Connecting with people online	Former users	−0.540	0.437	−1.235	0.58	−1.439, 0.285	0.28	1,133
	Resumed users	−0.543	0.650	−0.836	0.58	−1.950, 0.634		
	Discontinued users	−0.401	0.637	−0.629	0.67	−1.779, 0.759		
Connecting with a mental health professional online	Former users	0.141	0.400	0.351	1.15	−0.669, 0.906	0.22	1,131
	Resumed users	−0.826	0.795	−1.039	0.44	−2.730, 0.534		
	Discontinued users	0.899	0.505	1.782	2.46	−0.149, 1.851		
Use of professional services	Former users	0.297	0.398	0.745	1.35	−0.483, 1.080	0.43	1,151
	Resumed users	0.036	0.591	0.061	1.04	−1.146, 1.188		
	Discontinued users	−0.249	0.573	−0.434	0.78	−1.399, 0.870		

* *P* < .05.

However, three effects emerged. First, Discontinued Users (Current-Past Users) were more likely than Continued Users (Current-Current Users) to report use of other online mental health tools at timepoint 2 (39.7% vs. 21.2%), OR = 4.31, 95% CI = 0.508, 2.382, *P* = .002. Second, Discontinued Users (Current–Past users) reported higher impact of stigma on life (less stigma resistance) at timepoint 2 compared to those who continued use at both timepoints (Current–Current users), *b* = 0.38, SE = 0.17, *t*(1,114) = 2.25, *P* = .03. A third effect was observed for help-seeking stigma: Former Users, who were not using Headspace at either time point (Past-Past Users), reported less stigma towards help-seeking (better help-seeking perspectives) at timepoint 2, *b* = −0.27, SE = 0.12, *t*(1,115) = −2.33, *P* = .02. There was no effect of user type on connecting with others or a mental health professional online, or use of professional services, *P*s > .05. There were also no effects of user type or frequency of use on user experience scores at timepoint 2, *P*s > .05.

## 4 Discussion

This study examined whether individuals’ experience with and use of Headspace, within the context of a widescale deployment, affected mental health outcomes, mental health stigma, and utilization of other mental health resources.

### 4.1 Assessing improvements in distress, loneliness, stigma and mental healthcare utilization in a real-world setting

Overall, participants reported significant, yet extremely small, reductions in psychological distress and loneliness and increases in internalized stigma over four weeks. Although participants' baseline levels of distress and loneliness were moderate, the large sample size makes statistical significance less interpretable than the magnitude of change. The small effects are consistent with prior research on app-based mindfulness interventions, including recent randomized controlled trials (27, 28). For instance, Flett et al. (28) reported weak evidence for improvements in psychological distress following access to Headspace. Their intent-to-treat (ITT) analysis showed no significant differences between intervention and control groups, while per-protocol (PP) analyses indicated small improvements in distress for those who used Headspace, with effect sizes ranging from very small to medium. However, unlike Flett et al. (28), we did not find statistically significant results based on engagement. Our results show that both the paired *t*-tests and the regression analyses pointed in a consistent direction for distress and loneliness: small decreases over time with no significant differences by user type, and for internalized stigma: no user group differences but small overall increases. Importantly, our findings extend this evidence by showing that such reductions also occur in an uncontrolled, naturalistic setting, where distress decreased regardless of continuity or frequency of Headspace use. This aligns with prior research indicating that both brief and sustained mindfulness practice can yield mental health benefits (29–32). Given that our study relied on self-reported usage data, further research with objective app metrics is needed to clarify how the quantity and quality of app engagement influence mental health outcomes.

Overall, participants reported that healthcare utilization decreased over time, which may reflect reduced perceived need for support. While past research argues that DMHIs should complement rather than replace existing healthcare services (33), they can also reduce burden on healthcare systems by providing instant and 24/7 access to self-management support (34).

### 4.2 Discontinued users and use of other mental health resources

Interestingly, while overall use of other mental health resources decreased across the sample, Discontinued Users (those who used Headspace at timepoint 1 and stopped using it by timepoint 2) were significantly *more* likely to engage with other online mental health tools than those who continued using Headspace. The effect for Discontinued Users using other online tools was both statistically significant and can be practically meaningful. This finding indicates that discontinuing Headspace use may be related to some users seeking other digital resources for mental health support. This could reflect a desire for variety, different features, or alternative resources to address mental health needs (even if they had a positive experience with Headspace). It also might suggest that Headspace served as a useful front door for mental health support and that the experience led people to continue to seek out additional resources. People often use multiple tools to support their mental health, and may switch between them based on their needs at a given moment (35). The overall trend suggests people were using fewer resources, but Discontinued Users seem to be an exception, actively seeking alternatives after somewhat recently stopping Headspace. This supports prior findings that positive experiences with one mental health technology can facilitate uptake of others (36–38) or that those who stopped using Headspace somewhat recently may have found other digital resources better suited to their needs. This highlights Headspace's role within a broader ecosystem of DMHIs rather than as a standalone solution.

### 4.3 Former users and help-seeking stigma

The regression results, which considered different user groups (by comparing to Continued Users), suggest that engagement patterns, defined by user type and self-reported frequency, matter, revealing what may have been masked by looking at the overall sample with the paired *t*-tests. While a paired *t*-test across the entire sample showed no significant change in help-seeking stigma, which suggests that, on average, people did not experience a shift over time in their perceptions about getting support for mental health, the regression revealed that Former Users (those who were not using Headspace at either timepoint 1 or 2) reported lower scores on stigma towards help-seeking (help-seeking stigma) compared to Continued Users. Although the change is statistically significant, the magnitude of the effect is relatively small, which may indicate a small shift in attitudes toward help-seeking, suggesting Former Users are slightly less likely to hold negative views about seeking help for mental health concerns. This could mean that these individuals may feel less reliant on a specific mental health app for support and might have already developed alternative coping or management strategies (e.g., seeking professional help, using other resources, or relying on personal coping mechanisms). This is in line with previous research which found that higher stigma is associated with greater mental health app usage (39). Former Users' lower help-seeking stigma scores may reflect a lower need for anonymous and private support such as DMHIs. However, the modest nature of the effect means it might not substantially change behavior.

### 4.4 Discontinued users and stigma on life

Further, we observed a greater increase in impact of stigma on life (stigma resistance) among participants who stopped using Headspace within the previous four weeks. While Wang et al. (40) report that mindfulness is generally linked to lower stigma-related stress, our finding of increased impact of stigma on life (decreased stigma resistance) among consistent Headspace users might reflect a more complex, nuanced process. One possible explanation is that consistent Headspace users may have experienced a temporary increase in difficulty resisting internalized stigma or maintaining positive attitudes in the face of stigma. This heightened awareness might initially lower stigma resistance and increase the impact of stigma on life before mindfulness skills and coping strategies ultimately reduce stigma-related stress over time. This suggests future research to explore longitudinal work focused on dynamic evolution of stigma when using DMHIs like Headspace.

In practical terms, the change in stigma resistance among Discontinued Users may indicate that users who disengaged from Headspace might not have the same resilience to stigma in the mental health space as those who continued using it. Given that they had stopped using Headspace in the previous four weeks or less (compared with Former Users who had likely stopped using Headspace for over a month and Consistent Users), Discontinued Users may benefit from targeted interventions that address their potentially lower resistance to stigma and higher uncertainty after recently discontinuing tools like Headspace. This might involve reinforcing alternative mental health strategies or providing them with other resources to feel supported and less disconnected.

### 4.5 Strengths and limitations

A key strength of this study is its naturalistic design, assessing Headspace use in a real-world deployment outside controlled settings. This provides valuable insights into how users interact with the app in everyday life. However, this real-world context also introduces limitations, including less control over confounding variables. The study relied on self-reported usage measures, and objective usage data was unavailable, meaning engagement could not be independently verified. Self-reported usage measures may not fully capture the complexity, quality, or consistency of engagement. Additionally, in self-reporting usage, the majority (98%) of survey respondents indicated they had used Headspace, and there is no sufficiently large sample to compare results with a control group of participants that never used Headspace. The response rate for the initial survey was 3% and there is potential for response bias; care should be taken in concluding whether results were reflective of those who chose to engage with Headspace, or specific to those who completed the survey. The study also used parts of some scales in order to reduce participant burden. For example, while our stigma assessment aligned with the aforementioned collaborative community Headspace workgroup and the stigma conference with the subsequent factor analysis, it has its limitations in more holistically assessing such a nuanced construct. Lastly, the study duration was limited to four weeks and started during the COVID-19 pandemic. Changes may partly reflect broader contextual factors such as loosened restrictions in social distancing and stay-at-home orders. Future research should incorporate more objective and granular usage data to better understand the nuances of app interaction, what content was used, and its relationship to mental health outcomes, stigma, and mental healthcare utilization as well as to further investigate complex constructs using full scales whenever possible.

### 4.6 Conclusions

This study reported findings from a large-scale, naturalistic deployment of Headspace. Results from paired t-tests showed statistically significant changes over time in distress (decrease), loneliness (decrease), and internalized stigma (increase), but the magnitude of changes was small, likely limiting their clinical significance. Regression results showed some interesting and potentially meaningful user group differences in stigma resistance, help-seeking stigma, use of other tools. Perceived stigma and user experience were stable, suggesting no overall or user subgroup-level changes. Behavioral measures of mental healthcare utilization decreased overall, but Discontinued Users showed increased use of alternative tools, suggesting possible substitution effects in an ecosystem of digital tools used to support mental health. While findings suggest Headspace may act as bridge to other resources, its specific and full effects remain difficult to isolate and require further investigation using objective usage data and more nuanced measures of engagement quality and patterns.

## Data Availability

The datasets presented in this article are not readily available because the data collected and analyzed during the study are not publicly available as they were not funding requirements, and the study participants did not consent. Requests to access the datasets should be directed to Judith Borghouts, jborghou@hs.uci.edu.

## References

[1] Salaheddin K Mason B. Identifying barriers to mental health help-seeking among young adults in the UK: a cross-sectional survey. Br J Gen Pract. (2016) 66(651):e686–92. 10.3399/bjgp16X687313 27688518 PMC5033305

[2] Linardon J Cuijpers P Carlbring P Messer M Fuller-Tyszkiewicz M. The efficacy of app-supported smartphone interventions for mental health problems: a meta-analysis of randomized controlled trials. World Psychiatry. (2019) 18:325–36. 10.1002/wps.20673 31496095 PMC6732686

[3] King DR Emerson MR Tartaglia J Nanda G Tatro NA. Methods for navigating the mobile mental health app landscape for clinical use. Curr Treat Options Psychiatry. (2023) 10:1–15. 10.1007/s40501-023-00288-4 PMC10206563 37360961

[4] Lagan S D’Mello R Vaidyam A Bilden R Torous J. Assessing mental health apps marketplaces with objective metrics from 29,190 data points from 278 apps. Acta Psychiatr Scand. (2021) 144(2):201–10. 10.1111/acps.13306 33835483

[5] Camacho E Cohen A Torous J. Assessment of mental health services available through smartphone apps. JAMA Netw Open. (2022) 5(12):e2248784. 10.1001/jamanetworkopen.2022.48784 36576737 PMC9857226

[6] Goldberg SB Tucker RP Greene PA Davidson RJ Wampold BE Kearney DJ Mindfulness-based interventions for psychiatric disorders: a systematic review and meta-analysis HHS public access. Clin Psychol Rev. (2018) 59:52–60. 10.1016/j.cpr.2017.10.011 29126747 PMC5741505

[7] Wasil AR Gillespie S Shingleton R Wilks CR Weisz JR. Examining the reach of smartphone apps for depression and anxiety. Am J Psychiatry. (2020) 177(5):464–5. 10.1176/appi.ajp.2019.19090905 32354266

[8] Crockett MA Núñez D Martínez P Borghero F Campos S Langer ÁI Interventions to reduce mental health stigma in young people: a systematic review and meta-analysis. JAMA Netw Open. (2025) 8(1):e2454730. 10.1001/jamanetworkopen.2024.54730 39813031 PMC11736514

[9] Magid K Sagui-Henson SJ Sweet CC Smith BJ Chamberlain CEW Levens SM. The impact of digital mental health services on loneliness and mental health: results from a prospective, observational study. Int J Behav Med. (2024) 31(3):468–78. 10.1007/s12529-023-10204-y 37488324 PMC11106110

[10] Newby J Gupta S Hoon L Zheng W Whitton AE Huckvale K Brief digital interventions for psychological distress: an AI-enhanced response-adaptive randomized clinical trial. JAMA Netw Open. (2025) 8(10):e2540502. 10.1001/jamanetworkopen.2025.40502 41171275 PMC12579342

[11] Survey CHI. UCLA Center for health policy research. CHIS 2020 adult public use files (2020).

[12] Kozlov E Bantum E Pagano I Walser R Ramsey K Taylor K The reach, use, and impact of a free mHealth mindfulness app in the general population: mobile data analysis. JMIR Ment Health. (2020) 7(11):e23377. 10.2196/23377 33245289 PMC7732704

[13] Borghouts J Eikey E V De Leon C Schueller SM Schneider M Stadnick NA Characteristics associated with the use of the mindfulness meditation app headspace in a large public health deployment : cross-sectional survey study. JMIR Form Res. (2025) 9:e73457. 10.2196/73457 40844821 PMC12413571

[14] Abbott D Lack CW Anderson P. Does using a mindfulness app reduce anxiety and worry? A randomized-controlled trial. J Cogn Psychother. (2023) 37(1):26–42. 10.1891/JCPSY-D-20-00058 36787997

[15] Callahan C Kimber J Hu E Tanner L Kunkle S. The real-world impact of app-based mindfulness on headspace members with moderate and severe perceived stress: observational study. JMIR mHealth UHealth. (2024) 12(1):e52968. 10.2196/52968 38488513 PMC10986332

[16] Harris PA Taylor R Minor BL Elliott V Fernandez M O'Neal L The REDCap consortium: building an international community of software platform partners. J Biomed Inform. (2019) 95:103208. 10.1016/j.jbi.2019.103208 31078660 PMC7254481

[17] Harris PA Taylor R Thielke R Payne J Gonzalez N Conde JG. Research electronic data capture (REDCap)–a metadata-driven methodology and workflow process for providing translational research informatics support. J Biomed Inform. (2009) 42(2):377–81. 10.1016/j.jbi.2008.08.010 18929686 PMC2700030

[18] Kessler RC Andrews G Colpe LJ Hiripi E Mroczek DK Normand SLT Short screening scales to monitor population prevalences and trends in non-specific psychological distress. Psychol Med. (2002) 32(6):959–76. 10.1017/S0033291702006074 12214795

[19] Hughes ME Waite LJ Hawkley LC Cacioppo JT. A short scale for measuring loneliness in large surveys: results from two population-based studies. Res Aging. (2004) 26(6):655–72. 10.1177/0164027504268574 18504506 PMC2394670

[20] Russell D Peplau LA Ferguson ML. Developing a measure of loneliness. J Pers Assess. (1978) 42(3):290–4. 10.1207/s15327752jpa4203_11 660402

[21] Ritsher JB Otilingam PG Grajales M. Internalized stigma of mental illness: psychometric properties of a new measure. Psychiatry Res. (2003) 121(1):31–49. 10.1016/j.psychres.2003.08.008 14572622

[22] Griffiths KM Christensen H Jorm AF Evans K Groves C. Effect of web-based depression literacy and cognitive-behavioural therapy interventions on stigmatising attitudes to depression: randomised controlled trial. Br J Psychiatry. (2004) 185:342–9. 10.1192/bjp.185.4.342 15458995

[23] Corrigan PW Daniel Giffort P Rashid F Matthew Leary B Okeke I. Recovery as a psychological construct. Community Ment Health J. (1999) 35(3):231–9. 10.1023/A:1018741302682 10401893

[24] Sorkin DH Mukamel DB Eikey E V Mark G Schueller SM Schneider M Conceptualizing and measuring mental health stigma (2020).

[25] Venkatesh V Thong JYL Xu X. Consumer acceptance and use of information technology: extending the unified theory of acceptance and use of technology. MIS Q. (2012) 36(1):157–78. 10.2307/41410412

[26] Team RC. R: A Language and Environment for Statistical Computing. Vienna, Austria: R Foundation for Statistical Computing (2022).

[27] Schwartz K Ganster FM Tran US. Mindfulness-Based Mobile apps and their impact on well-being in nonclinical populations: systematic review of randomized controlled trials. J Med Internet Res. (2023) 25:e44638. 10.2196/44638 37540550 PMC10439468

[28] Flett JAM Conner TS Riordan BC Patterson T Hayne H. App-based mindfulness meditation for psychological distress and adjustment to college in incoming university students: a pragmatic, randomised, waitlist-controlled trial. Psychol Heal. (2020) 35(9):1049–74. 10.1080/08870446.2019.1711089 32046499

[29] Johnson S Gur RM David Z Currier E. One-Session mindfulness meditation: a randomized controlled study of effects on cognition and mood. Mindfulness (N Y). (2015) 6(1):88–98. 10.1007/s12671-013-0234-6

[30] Baer RA Carmody J Hunsinger M. Weekly change in mindfulness and perceived stress in a mindfulness-based stress reduction program. J Clin Psychol. (2012) 68(7):755–65. 10.1002/jclp.21865 22623334

[31] Howells A Ivtzan I Eiroa-Orosa FJ. Putting the “app” in happiness: a randomised controlled trial of a smartphone-based mindfulness intervention to enhance wellbeing. J Happiness Stud. (2016) 17(1):163–85. 10.1007/s10902-014-9589-1

[32] Economides M Martman J Bell MJ Sanderson B. Improvements in stress, affect, and irritability following brief use of a mindfulness-based smartphone app: a randomized controlled trial. Mindfulness (NY). (2018) 9(5):1584–93. 10.1007/s12671-018-0905-4 PMC6153897 30294390

[33] Berry N Bucci S Lobban F. Use of the internet and mobile phones for self-management of severe mental health problems: qualitative study of staff views. JMIR Ment Health. (2017) 4(4):e52. 10.2196/mental.8311 29092809 PMC5688247

[34] Koh J Tng GYQ Hartanto A. Potential and pitfalls of mobile mental health apps in traditional treatment: an umbrella review. J Pers Med. (2022) 12(9):1376. 10.3390/jpm12091376 36143161 PMC9505389

[35] Burgess ER Zhang R Ernala SK Feuston JL De Choudhury M Czerwinski M Technology ecosystems: rethinking resources for mental health. Interactions. (2021) 28(1):66–71. 10.1145/3434564

[36] March S Day J Ritchie G Rowe A Gough J Hall T Attitudes toward e-mental health services in a community sample of adults: online survey. J Med Internet Res. (2018) 20(2):e9109. 10.2196/jmir.9109 PMC5838359 29459357

[37] Chęć M Ligocka M Maciejewska M Samochowiec J Łodygowska E Samochowiec A. On-line psychological support in the evaluation of specialists and future specialists in Poland. Comput Human Behav. (2016) 64:703–9. 10.1016/j.chb.2016.07.015

[38] Jordan SE Shearer EM. An exploration of supervision delivered via clinical video telehealth (CVT). Train Educ Prof Psychol. (2019) 13:323–330. 10.1037/tep0000245

[39] Fürtjes S Al-Assad M Kische H Beesdo-Baum K. Mental health apps within the healthcare system: associations with stigma and mental health literacy. Arch Public Health. (2024) 82(1):126. 10.1186/s13690-024-01362-w 39152505 PMC11328358

[40] Wang Z Yip CCH Leung DCK Chan KKS. The impact of mindfulness on stigma stress and well-being among individuals with mental disorders. Mindfulness (N Y). (2023) 14(4):808–17. 10.1007/s12671-023-02111-w

